# Differences in Attack Avoidance and Mating Success between Strains Artificially Selected for Dispersal Distance in *Tribolium castaneum*

**DOI:** 10.1371/journal.pone.0127042

**Published:** 2015-05-13

**Authors:** Kentarou Matsumura, Takahisa Miyatake

**Affiliations:** Laboratory of Evolutionary Ecology, Graduate School of Environmental and Life Science, Okayama University, Okayama, Japan; CNRS, FRANCE

## Abstract

Individuals of both dispersal and non-dispersal types (disperser and non-disperser) are found in a population, suggesting that each type has both costs and benefits for fitness. However, few studies have examined the trade-off between the costs and benefits for the types. Here, we artificially selected for walking distance, i.e., an indicator of dispersal ability, in the red flour beetle *Tribolium castaneum* and established strains with longer (L-strains) or shorter (S-strains) walking distances. We then compared the frequency of predation by the assassin bug *Amphibolus venator* and the mating frequency of the selected strains. L-strain beetles suffered higher predation risk, than did S-strain beetles. L-strain males had significantly increased mating success compared to S-strain males, but females did not show a significant difference between the strains. The current results showed the existence of a trade-off between predation avoidance and mating success associated with dispersal types at a genetic level only in males. This finding can help to explain the maintenance of variation in dispersal ability within a population.

## Introduction

Dispersal is an essential issue for the survival of animals [1]. In many animals, dispersal carries potential consequences for gene flow across space; thus, individual dispersal distances serve as an important measure in predictive ecology [2–3]. Although dispersal is an elementary driver of ecological and evolutionary patterns, which factors are ultimately most influenced by variation in dispersal ability in a natural population remains unresolved. Dispersal ability is closely related to body form and reproductive success (reviewed in [4–6]). For example, in some insect species with wing size dimorphism, individuals that have longer wings and greater flight ability show lower reproductive success because more resources are invested in wing size than in abdominal size. On the other hand, short-winged individuals invest their resources in increasing abdominal size, and this increases their reproductive success [7]. Both dispersal and non-dispersal types are frequently found in natural populations due to a resource allocation trade-off, and this can help to explain the maintenance of variation in dispersal ability within a population [4–6].

Dispersal depends on the individual’s ability to move and includes walking. Therefore, dispersal behavior might affect the predation risk and mating success of individuals [8]. Dispersers may increase their mating success due to an increase in the rate of encounters with mating partners. However, the greater moving ability may also result in additional encounters with predators [9]. On the other hand, non-dispersers might reduce their rate of encounters with predators, which might increase their survival rates compared to dispersers with greater movement. However, the mate encounter rates of non-dispersers might be low, which could lead to reduced mating success.

The behaviors of predators and mates create large selection pressures on predator avoidance and mate choice [10–11]. Several studies have reported correlations between predation avoidance and mating behavior at the phenotypic level. For example, female guppies (*Poecilia reticulata*) prefer males that show more activity in the presence of a predator, but these males also suffer a higher risk of mortality due to predation [12]. Male fiddler crabs (*Uca mjoebergi*) that reemerge more slowly from their burrows after a predator attack experience lower predation risk but are also likely to suffer lower mating success due to lowered levels of activity above ground [13]. In the cases described above, we can see a trade-off between mating success and predation avoidance among individuals. Several previous studies have shown a trade-off between mating success and predation avoidance [14–15]. The trade-off cost may maintain variation in animal behavior. To maintain behavioral dimorphisms, a genetic basis of the behaviors is indispensable [16–17].

Artificial selection is an especially incisive method for the study of genetic trade-off costs [18–19]. Furthermore, this method can help to explain the maintenance of variation and the evolvability of a trait because it can reveal the benefits and costs to individuals with different survival strategies [17, 20–21].

In this study, we measured the walking distance of the red flour beetle *Tribolium castaneum* by using a moving distance analyzer (image tracker). Then, we artificially selected for walking distance for 15 generations. We established two strains of the red flour beetle with longer (L-strain) or shorter (S-strain) walking distances. We then compared the mating frequency and predation avoidance behavior of these strains using a predator, *Amphibolus venator*.

Beetles of the *Tribolium* genus are insects that inhabit primarily stored grains [22]. *T*. *castaneum* disperses by walking, and it seldom flies in storehouses. However, there has been no artificial selection experiment for walking distance in this beetle. A previous study showed a behavioral correlation between mating success and predation avoidance in a *T*. *castaneum* strain artificially selected for the duration of death feigning, i.e., tonic immobility [15]. Here, we compared the costs and benefits of mating success and predation avoidance between dispersers and non-dispersers in *T*. *castaneum*.

## Materials and Methods

### Insects

*T*. *castaneum* is known worldwide as an insect of stored grain. Its body is approximately 4–5 mm long, its developmental period is approximately 30 days, and the adults have relatively long lives, with a reproductive lifespan of 0.5–1 years [23–24]. *T*. *castaneum* are highly polygamous, and both sexes mate frequently throughout their adult lives [23, 25]. The mating behavior of *T*. *castaneum* can be easily observed (mean duration of a mating: 156.63 [mean] ± 20.29 [SE] sec, *N* = 19; K. Matsumura, unpublished data), and they do not exhibit male—male combat behavior or male courtship before matings [23].

The *T*. *castaneum* beetle culture used in this study has been maintained in the laboratory for more than 30 years according to the rearing method described by Suzuki and Nakakita [26]. The beetles were reared with a mixture of whole meal (Yoshikura-shokai, Tokyo) enriched with brewer’s yeast (Asahi Beer, Tokyo) as the rearing medium and were kept in a chamber (Sanyo, Tokyo) maintained at 25°C with a 16 h photoperiod (lights on at 07:00, lights off at 23:00).

*A*. *venator* is a predator of stored-product insect pests including *T*. *castaneum* and is often found in product storage facilities [27]. *A*. *venator* were collected from a grain store in Okinawa, Japan (no specific permissions were required for these locations/activities for the species used in the present study, and the field studies did not involve endangered or protected species) and reared using *T*. *castaneum* larvae as food in an incubator maintained at 27°C with a 16 h photoperiod (lights on at 07:00, lights off at 23:00).

### Artificial selection

Seventy-five virgin males and 75 virgin females (21–28 days old) were randomly collected from a stock culture, and each beetle was placed in a separate well of a 48-well tissue culture plate (Greiner Bio-One, Germany) with food. The next day, each beetle was gently placed on a piece of filter paper in a plastic petri dish (35 mm in diameter, 10 mm in height) using a brush. Before the experiment, each individual was kept for 2 h in the laboratory, which was maintained at 25°C, to adapt to the environment. Sixteen petri dishes per experiment were gently placed in an incubator maintained at 25°C, and walking distance was recorded for 30 min using a monochrome CCD camera (SEYE130SN, Science-eye, Japan). Walking distance analysis software (2D-PTV Ver. 9.0, Digimo, Japan) was used to measure the travel distance of each individual using the recorded image (30 min). The 10 males and 10 females with the longest walking distance (13.3%) were selected to propagate the long-walking strain (L-strain). Similarly, the 10 males and 10 females with the shortest walking distance (13.3%) were selected to propagate the short-walking strain (S-strain).

These 10 males and 10 females from the same strain (F0) were put together in a plastic container (70 mm in diameter, 25 mm in height) with food (20 g), and the females of each strain were allowed to lay eggs. From the offspring, 75 virgin male and 75 virgin female adults (21–28 days old) were randomly selected (F1), and their walking distances were measured again. As a control strain (without selection), 10 males and 10 females were randomly picked from the base population and selected to propagate (C-strain). This procedure was repeated at each generation for 15 generations. Three replicate lines were produced for the L-, S-, and C-strains. For the C-strain, walking distances were measured at the 10th and 15th generation. All strains were initiated at the same time and were kept in the same incubation conditions, i.e., all operations were carried out in an incubator at 25°C with a 16 h photoperiod (lights on at 07:00, lights off at 23:00).

To assess the direct response to artificial selection, we estimated the realized heritability of walking distance. Realized heritability was calculated as described by Falconer and Mackay [17]. Heritability was calculated as the regression of the population mean on the cumulative selection differential for the first 15 generations.

### Predation test

A virgin adult female *Amphibolus venator* was used as the model predator (*N* = 30). *A*. *venator* females were maintained individually in plastic petri dishes (35 mm in diameter, 10 mm in height) with *T*. *castaneum* larvae in an incubator (27°C with a 16 h photoperiod: lights on at 07:00, lights off at 23:00) until the day of the test. Before the test, each predatory insect (approximately 70 days old) was starved for 10 days.

The protocol of the predation test is shown in S1 Fig. An *A*. *venator* female that had been starved for 10 days was gently placed on a petri dish (60 mm in diameter, 15 mm in height) lined with filter paper and covered by a smaller petri dish (30 mm in diameter, 15 mm in height). Then, a *T*. *castaneum* virgin adult derived from either the L- or the S-strain (7–14 days old, from the 12th generation) was introduced into the petri dish (Predation test 1). Each individual was kept for 3 min before the experiment to adapt to the environment. After 3 min, we removed the smaller petri dish and then observed predation. The period from the start of the test until successful predation was timed with a stopwatch, and observation was carried out for up to 15 min. Individuals who survived predation for 15 min were recorded as 900 sec. Different beetles were used as prey in each trial, but the same predator was used for 6 predation tests (see S1 Fig). That is, different assassin bugs (*N* = 30) were used for different experiments (sex, strain, replicate line), and the order of these tests was fixed as follows (see S1 Fig): 1st male prey from the L-strain (replication 1), 2nd female prey from the L-strain (replication 2), and so on. We used 180 adult beetles of two sexes from the two strains, with three replications (*N* = 15 × 2 sexes × 2 strains × 3 replicate lines, see S2 Fig). In replication 1 only, an additional 6 females from the S-strain and 6 females from the L-strain were measured. Therefore, a total of 192 *T*. *castaneum* individuals were used in this predation test (L strain: *N* = 96, S strain: *N* = 96, see S2 Table). This design was not fully random, but all of the beetles selected from each replication of each strain should have encountered the different predators with almost the same probability. We also assumed that the duration of starvation (10 days) did not affect the experience and memory of the predator, although we must consider that the protocol might cause a slight degree of bias in the present results.

All experiments were carried out between 12:00 and 18:00 in a room maintained at 25°C with a 16 h photoperiod (lights on at 07:00, lights off at 23:00).

### Mating test

Fifteen virgin males (7–14 days old) were randomly selected from the 12th generation of each strain. To compare the mating abilities of the L- and S-strains, we measured the mating frequency of males from each strain for 15 min, using females of the C-strain as mating partners. Each individual was placed in a well of a 48-well tissue culture plate containing food until the time of the test. One day before the test, the elytra of the males were marked with paint (white, Paint Marker X -2, Tamiya, Shizuoka, Japan) to distinguish between the sexes during the observation of mating. We found no difference in the mating times of a female mated with males with and without paint, i.e., marking had no effect on mating time (females mated 3.3 [mean] ± 1.3 [SE] times when mated with painted males, *N* = 20; and 3.1 ± 1.1 times when mated with non-painted males, *N* = 20; *P* = 0.693, Mann-Whitney *U* test).

The next day, each male was placed gently in a petri dish (60 mm in diameter, 15 mm in height) that was inset with a piece of filter paper. Each individual was kept for 3 min before the experiment to adapt to the environment. After 3 min, five virgin females (7–14 days old, from the C-strain) were serially introduced into the dish, and the number of successful matings was recorded for 15 min (S2 Fig test for males). When a female mated with a male, she was immediately replaced with another virgin female derived from the C-strain. This procedure maintained a constant density of virgin females during the entire observation period. This experiment was replicated 15 times for each strain.

We carried out the mating test for females using the same method used for males. Virgin females (7–14 days old) of the 12th generation of each strain were randomly selected and placed in a well of a 48-well tissue culture plate containing food until the time of the test. One day before the test, we marked males (marking had no effect on mating, as described above). The next day, each female was placed gently in a plastic dish that was inset with a piece of filter paper. We waited 3 min for these females to adapt to the environment. Five virgin males (7–14 days old, from the C-strain) were then serially introduced to the female, and the number of matings was recorded for 15 min (S2 Fig test for females). When a male mated with the female, he was replaced with another virgin male from the C-strain. This experiment was replicated 15 times for each strain.

These tests were conducted solely to judge mating activity, not to judge female and/or male choosiness. All experiments were conducted between 12:00 and 18:00 in the room described above.

Although the selection was carried out for 15 generations, the mating test was conducted using beetles derived from the population of the 12th generation because we used beetles from the 12th generation of the control line. We also carried out the predation test at the 12th generation. Because we used populations from the same generation of the selected and control lines, we thought the current comparisons of predation and mating were as precise as possible.

### Statistical analysis

Analysis of variance was performed to test the significance of the realized heritability of walking distance. To test for the significance of the divergence due to selection in the 12th generation, walking distance was analyzed using a restricted maximum likelihood (REML) with selection regime (L-, S-, or C-strain) as a fixed effect and replicate line (1, 2, or 3) as a random effect. Predation rate, latency of predation, and mating success in the 12th generation were analyzed by REML with selected regime (L- or S-strain) as a fixed effect and replicate line (1, 2, or 3) as a random effect. If the REML estimates were significant in an analysis, we used Student's *t*-test (α = 0.05) as a post hoc test. All statistical analyses were performed using JMP version 7 (SAS Institute Inc. 2007).

## Results

### Direct response to selection

**Fig 1:**
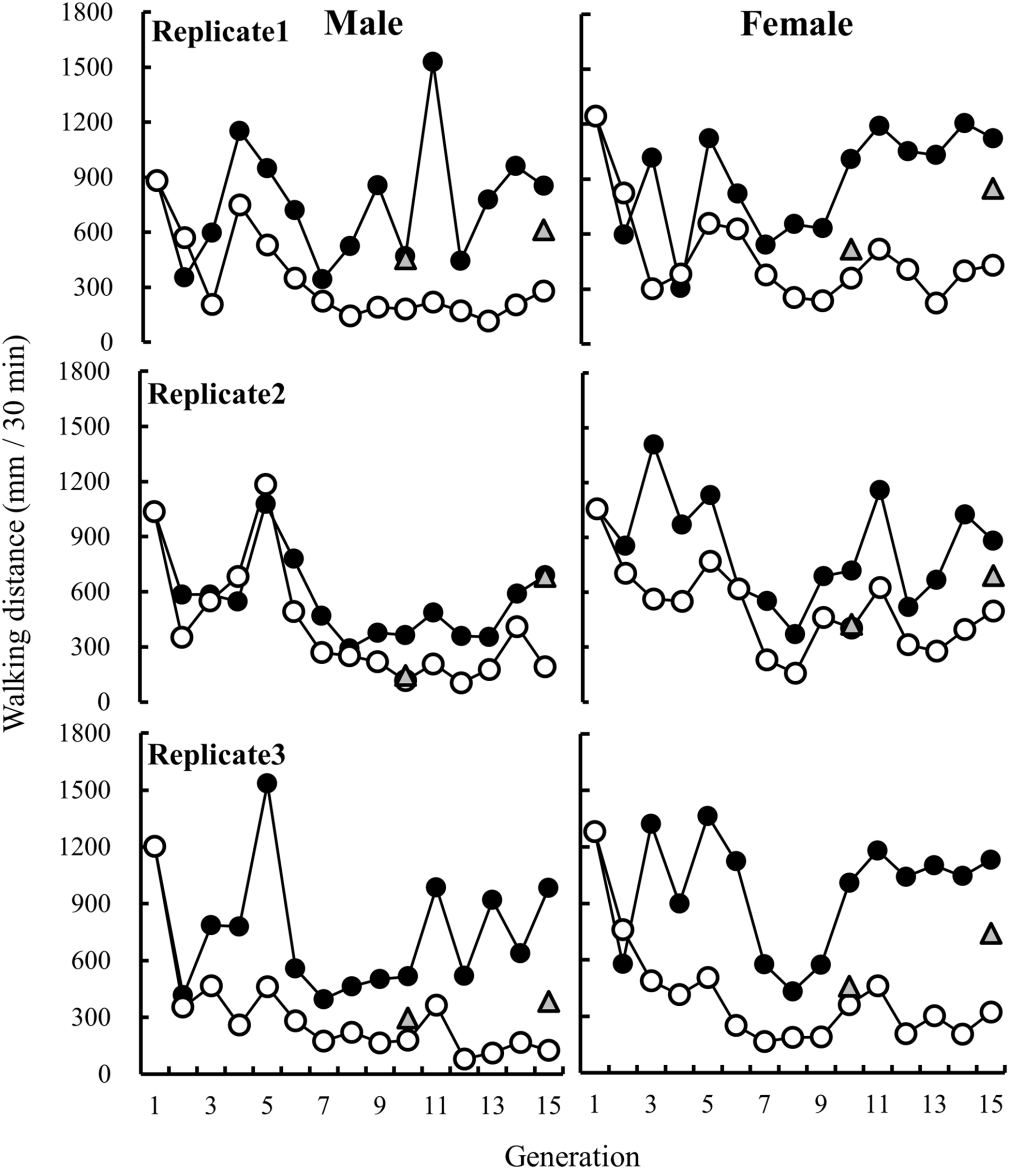
Direct response to selection for walking distance in *Tribolium castaneum*. Long (L) strains, filled symbols; short (S) strains, open symbols; control (C) line, triangular symbols. Upper, middle and bottom graphs show replicate lines 1, 2, and 3, respectively.

Fig 1 shows the direct response to artificial selection for walking distance over 15 generations. In all lines, the dispersal distances of the 12th generation in males and females of the L-strain were longer than those of the C-strain, whereas those of the S-strain were shorter than those of the C-strain (Fig 1 and S1 Table). Additionally, the dispersal distances of the 12th generation showed significant divergence between the L- and S-strains (strain: *F* = 171.79, *P* < 0.0001). Females traveled significantly longer distances than males in all strains (G12; sex: *F* = 25.97, *P* < 0.0001). No significant interaction effects between strain and sex were detected (G12; strain*sex: *F* = 0.22, *P* = 0.637).

Significant heritability of dispersal distance was detected in the S-strain, but it was not significant in the L-strain until the fifteenth generation (Table 1).

**Table 1:** Realized heritability (*h* ^*2*^) of walking distance in each strain.

Replicate line	Sex	*h* ^2^	
		Long strain	Short strain
1	male	0.015	0.152**
	female	0.019	0.096*
2	male	-0.002	0.128*
	female	-0.015	0.078*
3	male	-0.005	0.210**
	female	0.001	0.165**

Heritability was calculated as the regression of the population mean on the cumulative selection differential for the first 15 generations.

*P < 0.05,

** *P* < 0.01, (ANOVA)

### Reaction to predators

**Fig 2:**
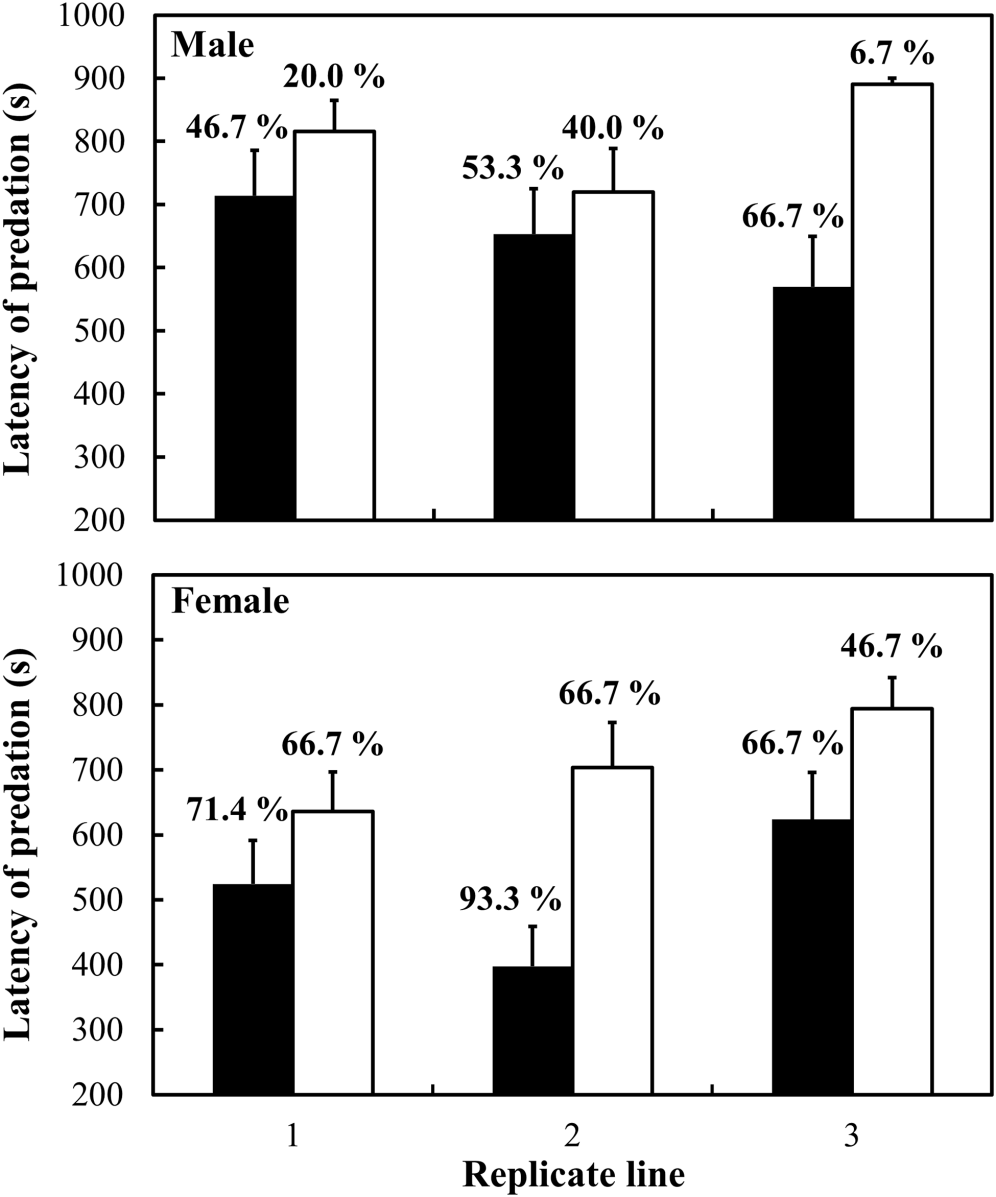
Latency of predation in selected strains (results of selection in the twelfth generation). Filled and open bars show the L and S strains, respectively. Error bars show SE. Predation rates are shown above the error bars.

Fig 2 (S2 Table) shows the predation rates (shown as a percentage in each box in Fig 2) and latencies of predation (shown as bars). The strains selected for long walking distance (L-strains) suffered higher predation risk compared to the strains selected for short walking distance (S-strains). The mean percentage of *T*. *castaneum* males preyed on by *A*. *venator* within 15 min was 55.6 ± 5.9% in the L-strains and 22.2 ± 9.9% in the S-strains. The mean percentage of females preyed on was 77.8 ± 0.09% in the L-strains and 60.0 ± 0.07% in the S-strains. Beetles from the L-strains suffered significantly more predation than beetles from the S-strains (strain: *F* = 13.58, *P* = 0.0003). Females suffered more predation than males (sex: *F* = 19.97, *P* < 0.0001), but there were no other significant interaction effects (strain*sex: *F* = 1.76, *P* = 0.1863).

The latency of predation within 15 min was shorter for the L-strains (males: 645.68 ± 74.28 sec; females: 516.26 ± 41.12 sec) than for the S-strains (males: 808.69 ± 42.48 sec, females: 702.35 ± 35.65 sec; strain: *F* = 13.58, *P* = 0.0003). Females were killed faster than males in all of the strains (sex: *F* = 19.97, *P* < 0.0001). No other significant interaction effects were found (strain*sex: *F* = 1.76, *P* = 0.1863).

### Mating success

**Fig 3:**
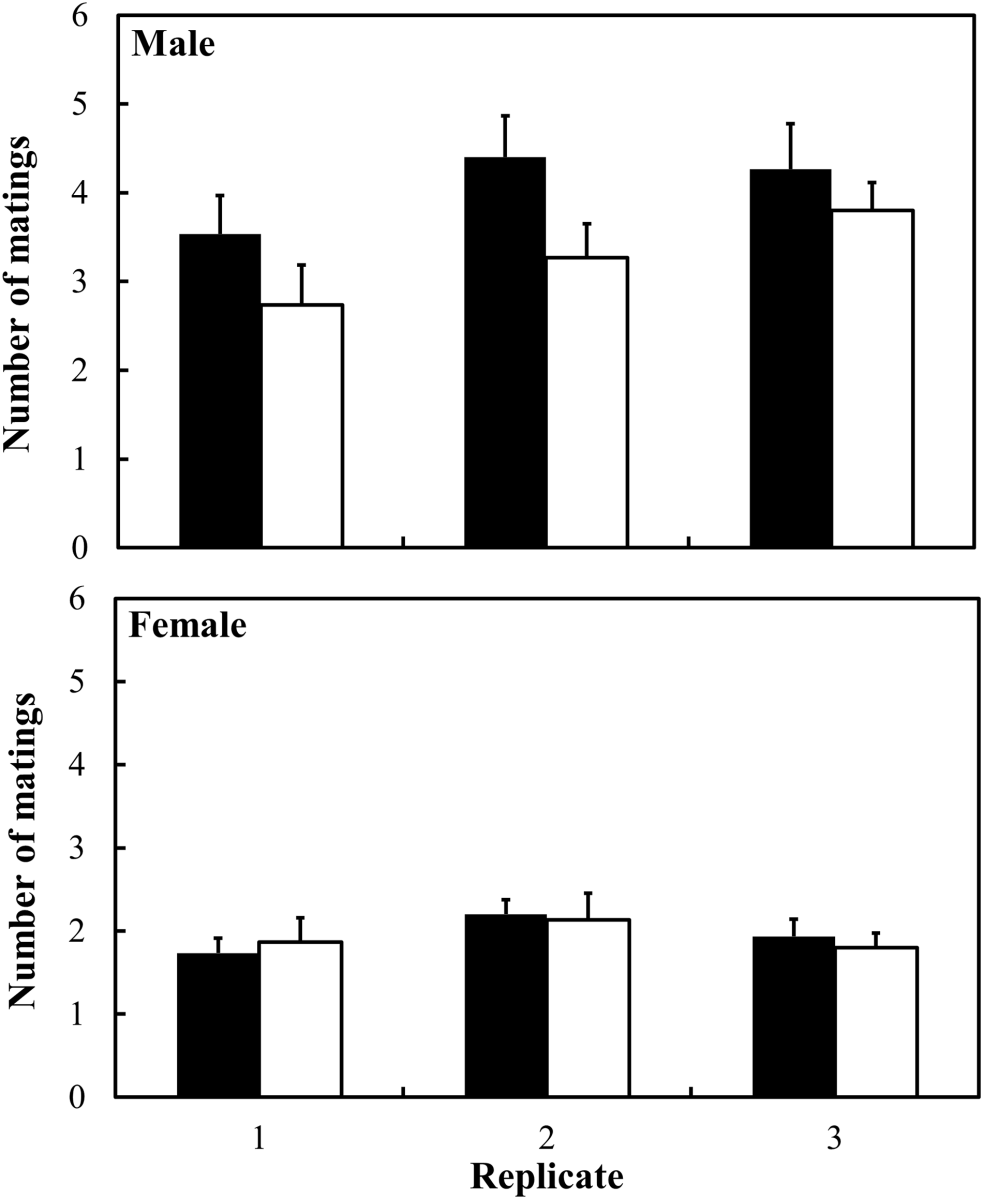
The number of matings in selected strains (results of selection in the twelfth generation). Filled and open bars show L and S strains, respectively. Error bars show SE.

Fig 3 (S3 Table) shows the number of matings for the selected strains. Mating success rates for 15 min were significantly different between the L-strains (male: 4.07 ± 0.47, female: 1.96 ± 0.14) and S-strains (male: 3.27 ± 0.38, female: 1.93 ± 0.10, strain: *F* = 4.27, *P* = 0.0403). Females had lower mating success than males in all strains (sex: *F* = 74.94, *P* < 0.0001), and no significant difference in mating success was found between the selected strains in females (Student’s *t*-test). No other significant effect was found (strain*sex: *F* = 3.82, *P* = 0.0522).

## Discussion

Artificial selection for dispersal distance in *T*. *castaneum* produced L-strain individuals who walked significantly longer distances than individuals from S-strains. Comparison of predation avoidance and mating success between the selected strains showed that L-strain males suffered higher predation risk by *A*. *venator* than S-strain males. On the other hand, L-strain males had greater mating success than S-strain males when they met C-strain females. The present results are consistent with our hypothesis that males with higher moving distances experienced an increased frequency of encounters with predators and mating partners than males with lower moving distances. Although the females derived from L-strains suffered a higher predation risk than those from S-strains, mating success did not significantly differ between the selected strains in females. Therefore, females with a genetically greater dispersal ability suffered a higher risk of predation but did not enjoy increased mating success.

Dispersal often affects the mating success of males. For instance, long-winged male crickets [28–31] and aphids [32–35] show a reduction in mating opportunities compared to wingless or short-winged relatives. These studies indicate that dispersal is a cost of increased mating success for males. However, the present results showed that males from strains selected for longer walking distances had greater mating success than males from strains selected for shorter walking distances in *T*. *castaneum*.

In *T*. *castaneum*, many studies for sexual selection have been conducted [36]. Pre- and postcopulatory sexual selection has been associated with reproductive success in studies using various strains and selection lines [37–43]. In the present study, however, only the number of matings was examined (see S1 Fig). Therefore, the present results cannot reveal whether female and/or male choosiness or postcopulatory selection was related to the selection protocol. Examining the relation between dispersal and sexual selection, including male and/or female choosiness and postcopulatory sexual selection, will be of great interest in *T*. *castaneum* in the future.

In the present study, no significant differences in female mating success were found between the selected strains. Therefore, the results suggest the absence of a relationship between mating success and dispersal in females. Previous studies have shown that multiple matings often have fitness costs for females in many insects [44]. In addition, *T*. *castaneum* females can use stored sperm for fertilization for over four months following a single mating [45]. Although females may proactively avoid multiple mating, which may reduce their fitness because of sexual harassment, female beetles are known to be able to gain sperm through multiple mating [36]. Therefore, future examination of the relationship between dispersal and the effect of mating system, including the pre- and postcopulatory costs and benefits, is necessary in the *T*. *castaneum* female.

In the sand cricket *Gryllus firmus*, which has dimorphism in wing size, the fecundity of long-winged females is substantially less than that of short-winged morphs [46]. These studies suggest that dispersal ability and fecundity reflect a trade-off. Similar results have been found in other studies [47–50], and these dispersal patterns can help explain the maintenance of variations in dispersal behavior [4–6]. The present study is the first empirical study of the relationship between predation avoidance and mating success focused on genetic dispersal ability.

Dispersal often increases the risk of predation (e.g., [51–57]). This phenomenon is due to the increased frequency of encounters with predators (see [8]). Actually, the present results also showed that individuals with genetically higher dispersal rates had a higher risk of predation than genetically sedentary ones. Thus, the present results are consistent with the results of previous studies [51–57] and strongly suggest that predation risk is a cost of dispersal. However, disperser males had higher mating success than non-disperser males. The negative correlation between predation avoidance and mating success is important because of the maintenance of dispersal variation (dispersers and non-dispersers) in wild populations of *T*. *castaneum*. In general, the majority of individuals travel short distances, whereas some travel much further [58], because dispersal is often associated with costs (see [8]). The present study showed that male dispersers accrued benefits and costs, but female dispersers accrued only costs. Consequently, the frequency of dispersers might be expected to be lower than non-dispersers in a natural population. Thus, this result may provide important information to explain the maintenance of polymorphism in dispersal types in natural populations. However, previous studies [42–43] have also revealed that the costs and benefits of multiple mating can be changed by environmental factors in *T*. *castaneum*. Therefore, we need to be careful to include information gleaned from the fitness data of both strains in future discussions of the costs and benefits.

Walking distances significantly diverged between the selected strains of *T*. *castaneum* due to artificial selection over 12 generations. Although a previous study carried out artificial selection for walking distance in the spider mite *Tetranychus urticae* [59], the results did not show a clear response to selection. Therefore, to our knowledge, the present study is the first empirical study to breed for divergent walking distance ability at the genetic level using artificial selection. However, significant realized heritability of walking distance was detected only in the S-strains, not in the L-strains. The base population of the selected strains was a long-husbanded laboratory stock whose selection by predators has been completely relaxed for hundreds of generations. If selection pressure from predators is absent for a long time, selection pressure might favor reproductive success due to the absence of a cost of predation. In the present results, males who walked long distances obtained the benefit of increased mating success, but females did not. Therefore, if dispersal distance is genetically correlated between males and females [60–61], dispersal ability in the stock culture might have already been shaped in a specific direction before this experiment through the male trait via a genetic correlation between the sexes. In other words, the genetic variance of higher dispersal ability in the study stock culture might have already been low; thus, artificial selection for longer walking distance produced no clear response. To test this hypothesis, artificial selection experiments based on field populations of *T*. *castaneum* may be required in the future.

In the present study, dispersal distances were artificially selected using a CCD camera (see methods). A previous study [62], which selected for the duration of death feigning, showed that the strains selected for shorter and longer durations of death-feigning exhibited longer and shorter walking distances, respectively, using the same method as the current study. The duration of death feigning has been artificially selected in a closely related species, *Tribolium confusum*, and the strains selected for shorter and longer durations of death-feigning showed higher and lower locomotor activities, respectively, demonstrated through the use of a device for recording locomotor activity [63]. However, the relationship between walking distance measured using a CCD camera and locomotor activity measured using a device for the recording of locomotor activity has never been examined. In the future, the relationship between the two parameters related to movement should be precisely examined in *T*. *castaneum*.

Nakayama and Miyatake [15] have shown that beetles derived from strains artificially selected for shorter and longer durations of death feigning suffered higher and lower predation risk, respectively, in *T*. *castaneum*. They also showed that males had higher mating success in strains selected for shorter rather than longer durations of death feigning [15]. Therefore, there is a possibility that artificial selection for the duration of death feigning [62] and for walking distance, as in the present study, involve a common genetic factor such as activity level, and thus cause a trade-off between mating success and predation avoidance in *T*. *castaneum*. The relationships between locomotor activity, walking distance and death-feigning behaviors should be examined in the future.

## Supporting Information

S1 Fig The protocol of the predation test. (TIF)

S2 Fig The protocol of the mating test. (TIF)

S1 Table Direct response to artificial selection for walking distance over 15 generations. (XLSX)

S2 Table Predation rates and latencies of predation. (XLSX)

S3 Table Number of matings. (XLSX)
