# Supplementary figures and images for: Differences in Attack Avoidance and Mating Success between Strains Artificially Selected for Dispersal Distance in Tribolium castaneum
Source: PLoS One. 2015 May 13;10(5):e0127042. doi: 10.1371/journal.pone.0127042 (PMC4430303; doi:10.1371/journal.pone.0127042)

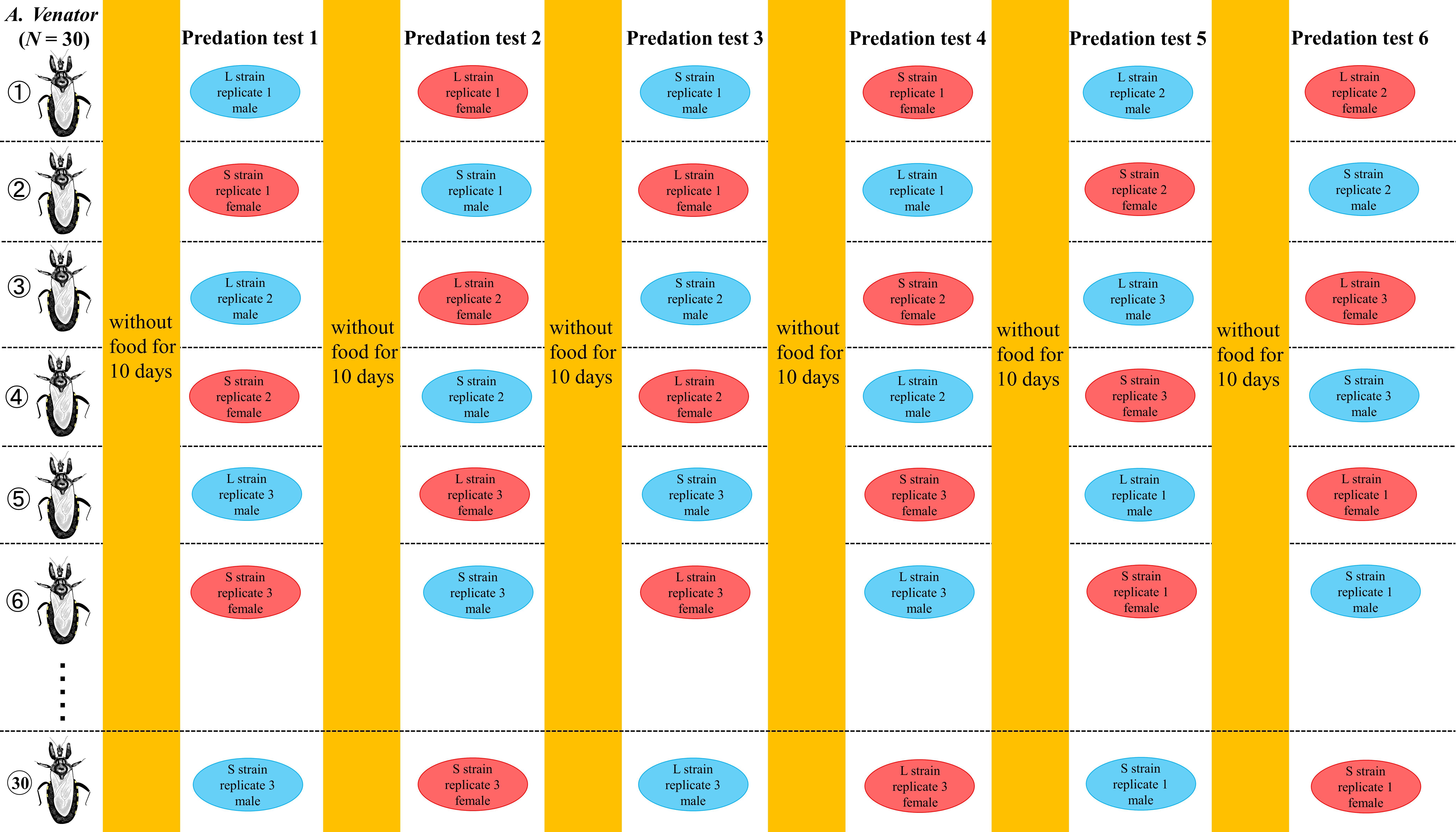

Supplement: S1 Fig — (TIF) [file pone.0127042.s001.tif]

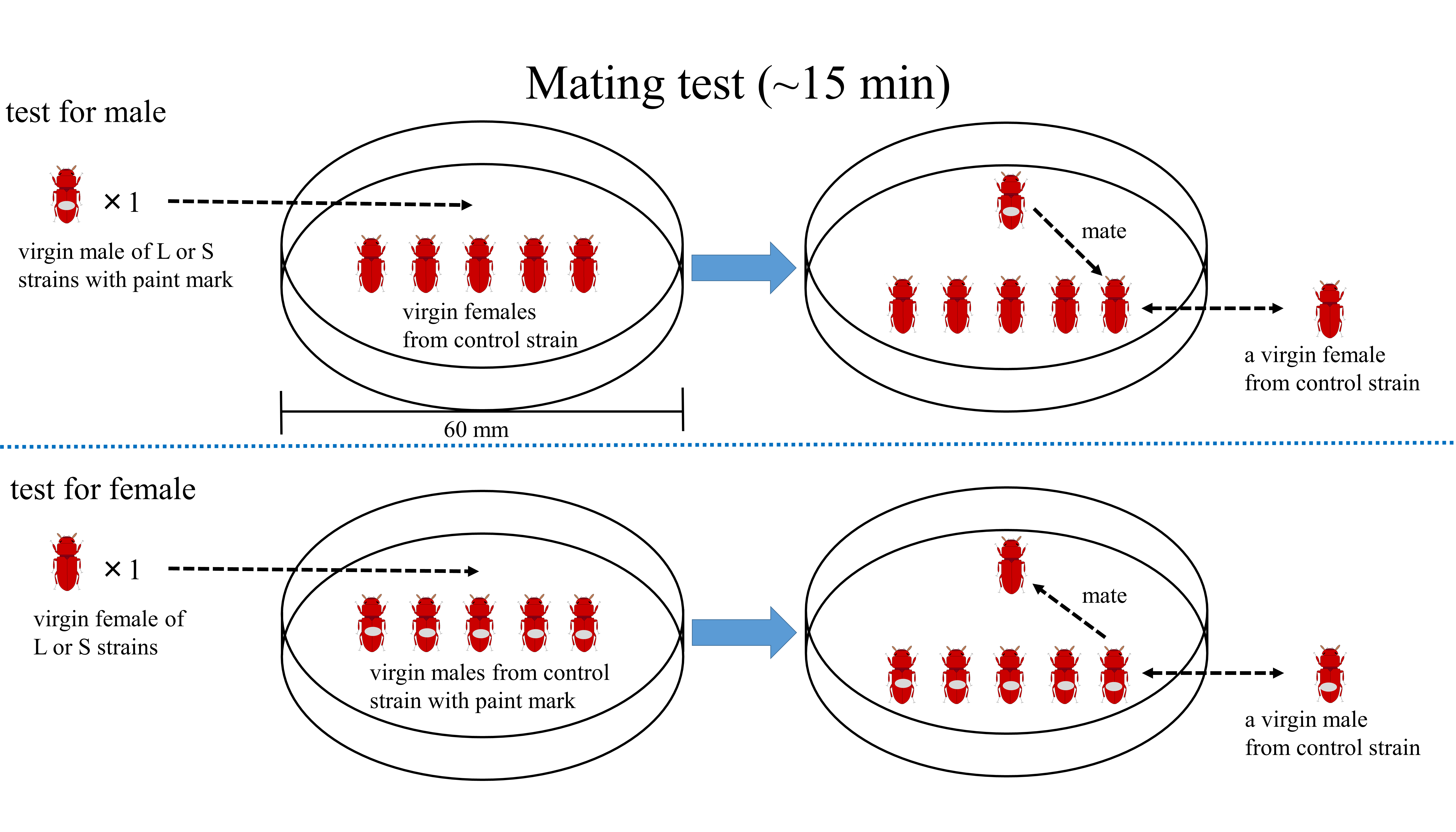

Supplement: S2 Fig — (TIF) [file pone.0127042.s002.tif]
